# The first mile: community experience of outbreak control during an Ebola outbreak in Luwero District, Uganda

**DOI:** 10.1186/s12889-016-2852-0

**Published:** 2016-02-16

**Authors:** Daniel H. de Vries, Jude T. Rwemisisi, Laban K. Musinguzi, Turinawe E. Benoni, Denis Muhangi, Marije de Groot, David Kaawa-Mafigiri, Robert Pool

**Affiliations:** Department of Anthropology, University of Amsterdam, Amsterdam, The Netherlands; Makerere University College of Humanities and Social Sciences, CeSSRA office, Kampala, Uganda; Department of Social Work and Social Administration, Makerere University, Kampala, Uganda; Center for Social Science Research on AIDS (CeSSRA), School of Social Sciences, Makerere University, Kampala, Uganda

**Keywords:** Ebola, Viral haemorrhagic fever, Outbreak control, Uganda, *Amayembe*, Spirits, Witchcraft, Community health resources, Anthropology, Luwero

## Abstract

**Background:**

A major challenge to outbreak control lies in early detection of viral haemorrhagic fevers (VHFs) in local community contexts during the critical initial stages of an epidemic, when risk of spreading is its highest (“the first mile”). In this paper we document how a major Ebola outbreak control effort in central Uganda in 2012 was experienced from the perspective of the community. We ask to what extent the community became a resource for early detection, and identify problems encountered with community health worker and social mobilization strategies.

**Methods:**

Analysis is based on first-hand ethnographic data from the center of a small Ebola outbreak in Luwero Country, Uganda, in 2012. Three of this paper’s authors were engaged in an 18 month period of fieldwork on community health resources when the outbreak occurred. In total, 13 respondents from the outbreak site were interviewed, along with 21 key informants and 61 focus group respondents from nearby Kaguugo Parish. All informants were chosen through non-probability sampling sampling.

**Results:**

Our data illustrate the lack of credibility, from an emic perspective, of biomedical explanations which ignore local understandings. These explanations were undermined by an insensitivity to local culture, a mismatch between information circulated and the local interpretative framework, and the inability of the emergency response team to take the time needed to listen and empathize with community needs. Stigmatization of the local community – in particular its belief in *amayembe* spirits – fuelled historical distrust of the external health system and engendered community-level resistance to early detection.

**Conclusions:**

Given the available anthropological knowledge of a previous outbreak in Northern Uganda, it is surprising that so little serious effort was made this time round to take local sensibilities and culture into account. The “first mile” problem is not only a question of using local resources for early detection, but also of making use of the contextual cultural knowledge that has already been collected and is readily available. Despite remarkable technological innovations, outbreak control remains contingent upon human interaction and openness to cultural difference.

## Background

### The first mile in disease outbreak control

There is general agreement that community members have a key role to play in early detection of Viral Haemorrhagic Fevers (VHFs) such as Ebola. Indeed, one of the greatest risks in the spread of VHFs is people lingering in the community without knowing they have contracted the disease [1]. At the start of the 2014 West African outbreak, Ebola spread unnoticed for three months [2]. Recent data suggest that the period of infectiousness in the community was five days on average, and at worst, more than 40 days [3]. Without effective isolation, it is estimated that each Ebola carrier transmitted the virus to between one and eight additional people, leading to a doubling time of around twenty days [4]. Early detection in the community presents a major challenge however, due to the difficulty for community members of recognizing nonspecific and common early symptoms such as fever, nausea, vomiting, diarrhea, and weakness. This is exacerbated by a general lack of local knowledge about biomedical explanations and by competing indigenous explanatory frameworks. The problem of how to engage local community members effectively in early detection when they remain typically outside the reach of the health system is reminiscent of an inverted scenario dubbed by disaster managers as the people-centered “last mile” of early warning [5, 6]. In the last mile, scientific information may be present about an imminent hazard such as an earthquake, but the early warning does not reach the communities on the ground in time, and is therefore futile. We suggest that the “first mile” of VHF outbreak control presents us with a comparable challenge in reverse: to try to capture timely information about an imminent outbreak from the community on the ground.

To remedy the first mile gap, investment is focused increasingly on technologies that accelerate bottom-up information provision from communities to public health level. Location-tagged community level mobile reporting systems are linked directly to national command units and international emergency aid agencies [1]. Uganda’s E-health infrastructure, for example, provides a foundation for “real time information sharing”, where information is distributed instantaneously [7]. This is often seen as the best solution to the first-mile problem: “With smartphones in the hands of all community health workers, drivers, facility managers, and district health officials, it should be possible to create a map of the epidemic in real time” [4]. Yet, as in the last-mile problem – where investment in high technology fails to reach vulnerable populations at risk – technology alone may fail to solve the first-mile problem if community members are not engaged. The real-time ideal collapses when those marginalized from the development process are left out of the community detection process [5, 6].

Can a more “people-centered” early detection system also be achieved? The standard response to this question is to suggest an enhanced role for community health workers who should be “quickly trained and deployed to identify people in the community who develop suspicious symptoms” [8]. Secondly, a “social mobilization” agenda is often called for, where entire communities participate in “quickly identifying and isolating infected individuals before they can transmit the virus” [9] – or, as the World Health Organization plainly puts it, “involve everyone” [10]. Literature acknowledges that community health worker and social mobilization strategies for the facilitation of rapid outbreak detection should be combined with culturally appropriate community-based trust building [11, 12].

In this paper we ask to what the extent the community became a resource for early detection and outbreak control during the critical initial stages of the epidemic, when risk of spreading was at its highest (“the first mile”). We identify problems encountered with community health worker and social mobilization strategies.

### Indigenous beliefs and Ebola

Indigenous representations of local responses to VHFs in under-resourced contexts such as rural Africa often include images of ignorance, exoticism and superstition, with “risky traditional burial practices” typically cited to illustrate the community as barrier [12–14]. As anthropologists have argued for decades, popular simplifications of community relationships create the impression that the local is stagnant, illogical and ignorant, while representations of international health communities tend to overemphasize collaboration, cooperation and organization [15]. Previous to the 2014 West African outbreak, the anthropologists Hewlett & Hewlett argued that representations of Ebola in the media are seldom contextualized; “one is left with a feeling that an outbreak is controlled only through Western biomedical knowledge and technology, in spite of, not because of, the actions of local peoples” [16].

Unfortunately, for the international health response to Ebola, only a few case studies are available in published literature detailing how such an outbreak is locally understood, responded to, and embedded within community relationships. This may be because anthropologists were invited late to participate in fieldwork relevant to VHF outbreak control [16, 17]. The Hewletts published the first anthropological account of indigenous reactions to Ebola 25 years after the first reported Ebola outbreak; they were invited only after cases continued to increase during Uganda’s 2000 outbreak, due to perceived “problems with local people” [16]. The Hewletts reported that American and Italian physicians working in isolation units had no idea how local people explained or viewed Ebola, while local northern Uganda health workers appeared unwilling to bring local cultural beliefs to the fore in an international healthcare response context. As the Hewletts wrote: “They feared that others hearing them talk about local beliefs would view them as backward, primitive, and exotic” (p41).

Investigating the local cultural beliefs of northern Uganda in the 2000 outbreak, the Hewletts documented *gemo* as a “bad” spirit that arrives suddenly and causes a mysterious illness within a very short time. They observed that *gemo* was associated with prescriptive behaviors and protocols for isolation of suspected cases, as well as burial at the edge of villages; it was common knowledge to children who had not learned about it in school. Further, in both the Congo and Uganda cases studied, they showed that as deaths continued despite indigenous treatments, people shifted their explanatory models from community illnesses to biomedical models. From this perspective, the Hewletts and others have argued for more respect and understanding and stressed the central importance of trust and rapport with the local community [13].

### Ebola in Luwero

This paper investigates this trust and rapport more than a decade later, in the same country that the Hewletts worked in. After Sudan Ebola was confirmed in Luwero County, the Ministry of Health set up a District Task Force (DTF) with case management, surveillance, ecological, psychosocial and social mobilization teams. In Luwero, the International Federation of Red Cross and Red Crescent Societies (IFRC) funded and organized social mobilization and psychosocial support. The IFRC pledged to produce and disseminate context-specific information, education and communication materials (38 K posters, 50 K leaflets and 120 T-shirts), conduct media campaigns (5 talk shows and 400 radio spots), and conduct one field media visit [18]. The IFRC enlisted and trained local volunteers and local community health workers (VHTs) to provide psychosocial support and built community trust and confidence. According to the evaluation, this process was successful and “put to a halt the spread of the disease and limited mortality as well as contributed to the well-being of the community” [19] (P16). The evaluation claimed that sensitization measures dissolved any myths or misconceptions, influenced behaviors and practices and provided psychosocial support, while information flow enhanced visibility and public confidence in response activities [19].

How did community members understand and experience the disease, and the external interventions that arrived in its wake? Did the community become a resource and partner – instead of a “barrier” – to outbreak control efforts, effectively bridging the first mile gap?

## Methods

The Ebola outbreak occurred in Kakute village in Ssambwe Parish (population 9400), just 30 minutes by car from our main field site. We were conducting 18 months of ethnographic research in a number of villages in Kaguugo Parish (population 4000), part of Luwero district, 75 km north of Kampala. As well as proximity, our main field site was similar to Kakute village in terms of rurality, Buganda culture and socioeconomic status.

Two months after Ssambwe Parish was declared free of Ebola, on January 16, 2013, one of our Ugandan doctoral researchers visited Kakute village to conduct interviews with an Ebola survivor, the DTF Chair, village chair, two nurses at isolation units, and one neighbour of the involved family. These key informant interviews were complemented by a focus group discussion comprising seven Kakute village participants. A total of 13 Kakute respondents were interviewed. The informants in Ssambwe Parish were chosen through non-probability “snowball” sampling. A snowball sample is a non-probability sampling technique in which the researcher collects data on the few members of the target population that he or she can locate, then asks those individuals to provide information to locate other members of that population whom they know. Snowball sampling is appropriate to use when members of a population are difficult to locate, but is not likely to provide a representative sample. In this case, snowball sampling was used because outbreak messages to the community had warned village residents to distance themselves from possibly infected residents and their “contacts”. As a result, key respondents had to some extent “gone underground” with respect to their beliefs. Our researcher traced the infected family and community members who had played a significant role influencing the community’s Ebola history. In addition to this targeted fieldwork in Ssambwe Parish, data from ethnographic research in nearby Kaguugo Parish by all three Ugandan doctoral students was used for this paper. Our team observed and documented the outbreak response control measures, and spoke about Ebola with an additional 21 key informant interviews and 61 focus group respondents. Table 1 summarizes the total number and location of all respondents.

**Table 1:** Number of key informants and focus group respondents by location and Parish in Luwero County

Location	Parish	# of key informants	# of focus group respondents	Totals
Bukuma	Kaguugo	3	8	11
Bunyenye	Kaguugo	0	16	16
Dekabusa	Kaguugo	5	15	20
Kakute	Ssambwe	6	7	13
Undefined	Kaguugo	3	8	11
Kyetume	Kaguugo	2	0	2
Ssakabusolo	Kaguugo	2	7	9
	Totals	21	61	*82*

All interviews were held in Luganda, the native language of the Baganda, spoken by the doctoral researchers and their Baganda assistants. Interviews were translated into English. Interview questions were open-ended. No specific standard interview instrument was designed for the purpose of documenting Ebola. Questions were developed in dialogue, guided by the ethnographic experience of the interviewers. All interviews and focus groups were audiorecorded, transcribed and analysed using the NVivo qualitative software analysis program. Textual information was analysed using grounded theory. Through review of collected data, repeated ideas, concepts or elements became apparent, which were tagged with codes and extracted from the data. These codes were grouped into thematic clusters. This resulted in the following themes: 1) critical discourse, 2) emergency response, 3) history of spread in Luwero, 4) perceptions of Ebola, 5) post-Ebola normalization, and 6) sources of information. These themes are further explored and organized below, divided into major subthemes summarizing the communal and indigenous experience.

Limitations to this method include subjective bias that is always part of qualitative study. In this case study, a major limitation is the short length of time we were able to spend on the ground in Kakute community. We also only had opportunity to interview a relatively small number Kakute residents, even though the interviews lasted long enough to obtain needed insight. However, we feel strongly that the ethnographic understanding provided by three separate ethnographers in nearby Kakuugo Parish brings in-depth substantiation of prevailing community attitudes. We observe little cultural difference with nearby community of Kakute. Further, while qualitative and/or ethnographic studies are also limited in generalizability, results provide conceptual frames of reference that help understand dynamic processes otherwise not easily captured using statistical methods.

The manuscript was reviewed by all authors, and further insights were added, arising from on-going dialogue in weekly group meetings of the research team. Ethical clearance was obtained from the School of Public Health at Makerere University College of Health Sciences (Reg. Number 158) and Uganda National Council for Science and Technology (Reg. Number SS 2754). Written informed consent for participation in the study was obtained. Observation was conducted with the approval of village chiefs. No children participated in the study.

## Results and discussion

### The outbreak

The Ebola outbreak in Luwero can be traced to a 32-year-old Islamic Kakute village motorbike taxi rider called Kabugo. His illness began with a fever in October 2012, which his family initially took for malaria. He suffered for a week before being taken to a health center. After a week of treatment, he spent a night at home where his family saw that he was still not well, so they took him to the hospital at the local army barracks. There, he started vomiting blood. The hospital staff shifted him to an isolated ward and took blood samples to establish the cause of his illness. Kabugo died shortly thereafter, before the results from the tests could be relayed to the family. During his Muslim funeral, his body was laid for final viewing during the night, and relatives and community members touched and washed his body. He was buried the following day according to tradition. During his funeral, Kabugo’s widow Halima developed similar symptoms and was admitted to the health center. She was attended by her mother-in-law, two sisters in-law, and an aunt who spent the nights with them and left early in the morning to attend to her business. The health staff at the clinic suspected that the widow had malaria, but as she tested negative the doctor concluded it was depression because she had lost a husband, and allowed her to be discharged. Her father took her to a traditional healer to investigate the mysterious cause of her husband’s death. She died shortly after. Two days later one of the sister-in-law developed symptoms and died. Two days after that, the second sister-in law died.

Staff at the health center started to suspect Marburg disease, and samples obtained from the patients were shipped to the Uganda Virus Research Institute. When these tested positive for Sudan Ebola, the Ministry of Health declared an Ebola outbreak on November 16, 2012, and established an isolation center. By November 28, there were six confirmed cases and one probable case, including four deaths (two from the same family), in Luwero and Kampala [19]. Twelve suspected cases tested negative. Medical personnel noted that their experience of earlier outbreaks of Ebola and Marburg in nearby regions helped them to deal with this outbreak; measures included pooling staff at national level from other parts of the country, extra training, mixing experienced staff with new staff, and provision of psychosocial support for medical staff. One medical respondent noted:

In Gulu over 200 people died; in Bundibugyo, over 30 people died; in Kibale about 17 died. So what I could ultimately say is that controlling the epidemic in a short time with minimal loss of life is the best thing we gave to the community. It might not be easily visible to some people, but as part of the system I think that was a very big achievement or reward. It is the lowest number.

One DTF member said that as death in Africa can sometimes be attributed to witchcraft, the main focus of the social mobilization team was to strengthen the community component which delivered the “actual definition by the health system of the problem, and how it can be prevented”. The member noted that to convince the community, the outreach response team used a clinical presentation that focused on clinical symptoms in order to dismiss ideas of witchcraft. The team’s main aim was to change people’s behaviour quickly:

And what you are aiming at in an epidemic is how to change people’s behaviour very fast. So we aim primarily at changing behaviour to control the spread of the infection. All the other things come later.

This behavioural change was generally perceived to be a “fight against misconception”. According to our DTF respondent, this fight was won by the social mobilization team, which succeeded in getting the facts across. “That is why we were able to contain it. We lost only five people from the same village, the same family.”

### Spirits

However, in Kakute, there was little evidence to support the idea that social mobilization had been a success. The majority of villagers continued to attribute Kabugo’s illness to witchcraft by his mother. One community member noted:

There is nobody in this village who believes that it was Ebola. The people did not even think of any other name for the disease because they were sure that it was *amayembe*.

Respondents noted that even the local motorcycle (*bodaboda*) boys who had worked with Kabugo did not believe that he had died of Ebola. The local explanatory framework supporting this interpretation was in place before Ebola was diagnosed; when Kabugo fell ill, a traditional healer argued that someone had bewitched him. It was said that when Kabugo and his kin sought charms, called *mayebe*, to remedy the witchcraft, one of his brothers refused to be involved, fearing that the spirits would mix with evil spirits – *kifaluu amayembe* – and cause the whole family to die. His fears seemed to be substantiated. His father, accused of witchcraft, was murdered; his own brothers among the killers. Upon his death, Kabugo collected his father’s spirits, and unable to meet their needs, became mortally ill. A local traditional healer then warned that his death would be followed by many more in his family. Stories were also circulating that Kabugo’s mother-in-law had used evil spirits to kill his wife, with whom she had had an acrimonious relationship. Community members asserted that in one of her many quarrels with Kabugo’s wife, the mother-in-law brazenly undressed and showed her bare buttocks, a sign that she cursed her son’s family. They also believed that when Kabugo’s body was brought home, a bat entered the house and disappeared into the body of one of his sisters. The community accused his mother of using *amayembe* to kill her children and obtain their property. After Kabugo’s death, community members went to her home to burn her house down and demand that she leave the village. Although her husband was able to prevent the burning, she did leave. Members of the Ebola team got wind of this event, found her on the road and took her to Mulago Ebola Response/Treatment center in Kampala for monitoring.

Community members believe in various types of *amayembe*: some benign, such as sources of fertility, and others malicious (*kifaluu amayembe),* used if a person has ill feelings towards someone, or is simply greedy and enlists supernatural forces for their own gain. Moving around at night, these spirits “eat” people and animals by sucking blood, knocking on doors, and raping women. When out of control, however, *amayembe* may turn against their buyers, their families, and sometimes beyond, particularly if their owners fail to provide sufficient sacrifice, in the form of goats or people, in return for the wealth that has been promised. In stories, community members describe seeing *amayembe* or their traces. For example, a 25-year-old Kaguugo man vividly described how his whole town went “on fire” when the local healer who came to deal with the *amayembe* fell out of a tree while chasing after it: “We thought he had broken his legs, but he would run while hitting his hands as flames would be seen, and there he was chasing the *amayembe*.” The idea of greed often plays a role in the *amayembe* narrative, suggesting that the concept represents an embodied rationalization of paranoid suspicion [20] and a social leveling force, both undermining and facilitating inequalities in wealth and power [21]. One respondent related the emergence of *amayembe* to a time when there was a lack of unity in the local community because “some people didn’t behave well”. *Amayembe* also trigger social isolation. Those who get in trouble are not only punished (through illnesses) by the *amayembe*, but they are also ostracized by the community. This is essentially an outbreak prevention strategy. A Kaguugo villager noted:

You see there are those who buy *amayembe* in this area. Now, if those *amayembe* kill people in the village, we give up on such a person in the village. We stop dealing with such a person. So it means that even when such a person loses someone, he has to care for his own needs (*Tumusamu*).

*Amayembe* may also be thought of as a system of belief that falls outside governmental control because – as many community members noted – the government does not acknowledge its existence. *Amayembe* are the domain of traditional healers at odds with a biomedical public health system. As a Kaguugo community member said:

So the greatest challenge is that when you report that people are dying because of *amayembe* they will ask ‘what is *amayembe?’* and you really cannot prove their existence. So that is why many people are dying and the government cannot do anything.

### The difficulty of dispelling *amayembe*

The *amayembe* interpretative framework described above leads to a credibility problem for biomedical explanations, which are seen as unclear and illogical interpretations of the genesis of particular outbreaks in the context of traditional aetiologies. This is not to say that the community was entirely united in this perception. Some community members likened the disease to smallpox (*kawumpuli*). But the belief that the disease was *not* Ebola was corroborated by logical observations made by the majority of the Kakute community and affirmed by Kaguugo locals. Crucially, the deaths occurred only in one family, and specifically affected people who the mother did not like. This despite the large number of people who had been in physical contact with the first patient, including the traditional healer. It was simply seen as unbelievable that a very infectious disease would not affect any of these other people. Furthermore, Kakute villagers argued that while health workers stated that Ebola causes victims to bleed from every pore and die within 24 hours, patients in Kakute lived for at least a week, did not bleed from every pore, and did not always suffer from high temperatures. In addition, it was locally known that previous Ebola outbreaks, in particular that in Kibaale the same year, had also primarily affected members of the same family (13 out of 17) [22]. Luwero residents further argued that the public health authorities had not connected the case of those who died in Kibaale with “eating infected animals”, a warning which figured prominently in the information circulated to the community, and there was no evidence that Kabugo or any of the other victims in Luwero had eaten wild animals such as monkeys before falling ill.

To confirm whether this disease was indeed Ebola, community members monitored one female neighbor who had interacted heavily with the diseased victims, as her house was surrounded by those of the family involved. Community members said that “even if she had just got a headache they would all scamper for safety, knowing that it was indeed Ebola.” This case was a yardstick for the community, and because she did not suffer any health problems, community attitudes did not change. The woman herself believed that God had saved her. Locals began to call for traditional healers to come and investigate exactly what had attacked the community. As a Kakute community member noted:

Even when the health workers came, the people asked them if they had considered investigating if these could be spiritually instigated attacks. They suggested that since we had used side A [medical approach] and people continued to die, we should switch to side B [traditional healers] to see if the problem could be properly diagnosed. But this was not done because the authorities insisted that people should accept that this was Ebola.

Community members asked the district chairman to send traditional healers to make their assessment of the situation. But this was stopped by police intervention, to prevent revenge killings of those villagers who might be identified as spreading *amayembe*. A Kakute resident noted:

The head of Police in the district said that he would arrest anyone who promoted the idea that the deaths were due to *amayembe*, and threatened to take stern action if anything happened to those suspected of bringing *amayembe*. He read out some names of those who had been listed as promoting this thinking among the community, and people got scared of discussing it much.

By the time the health authorities ruled that people should avoid attending large gatherings, the community was still preoccupied with ways to establish whether *amayembe* was to blame*.* Gatherings continued with public testimonies, one of which involved a daughter from the affected family “testifying” that her mother had *amayembe.* Community members said that according to the daughter, the spirits had “asked for the lives of seven family members and thirty from outside the family after the seven had died.” This led to increased fears within the community. One resident explained that he fled the village when the seven Ebola cases were about to be reached, for fear that he would be among the thirty who would subsequently be sacrificed from *outside* the family. Ironically, because people did *not* continue to die, this discourse strengthened local conviction that it was indeed, after all, *amayembe*, and not Ebola.

### The community as barrier to outbreak control

When Ebola outreach workers tried to provide relief items to the children of the husband of one of the female Ebola victims, he refused them entry unless they admitted that his wife died not of Ebola, but because of *amayembe*. Such resistance to medical interpretation was widespread. The IFRC evaluation report noted a “strong negative community reaction”, which eased over time, but did not disappear. One community member belonging to the social mobilization team noted:

It was like talking to mad people. They were angry, with some of them totally rejecting the message, while some agreed, but we did not really change the thinking of many that it was not *amayembe*. Generally the outbreak did not change the cultural beliefs of the people, and the people were not scared to talk to medical personnel who had touched patients.

One of the surviving Ebola victims described how he was returned to Kakute village in an ambulance because the doctors wanted the villagers to see that the survivors had indeed been treated at the hospital, and not by traditional healers. But community members shouted that the survivors had bribed the medics to say it was not *amayembe* and they argued that he had survived Ebola by escaping from Mulago to visit a traditional healer back home.

Eventually, the community’s resistance to biomedical interpretation was met with government oppression. A Kaguugo district community leader told us that while communities were disputing health workers’ claims about Ebola, district level government officials arrived with armed law enforcement to convey the seriousness of the issue. One Kakute respondent noted:

They warned that anyone who continued insisting that this was not Ebola would be dealt with according to the law. So that is why people started to listen quietly and behave [laughs]. So we needed that kind of authority to sit and listen to the health workers. We attended those sensitization sessions through the barrel of a gun. Before the government used force, people would come to the sensitization sessions, argue with the health workers and disrupt their sessions. But when the guns came in they had to sit and listen.

Even the youths, “the most critical audience within the village”, were forced to attend and listen, but it is unlikely that this strategy changed their minds. Rather, it reinforced historical mistrust of a chronically underfunded medical system.

Community members themselves provided hints in our interviews of how trust could have been built if the approach had been different. They argued that much could have been achieved had the response been speedier, with a concerted effort showing the community that the medical system actually cared for their thoughts and wishes. After confirmation of Ebola, our respondents noted that social mobilization came too slowly to counter the growing *amayembe* interpretation effectively. This delay in outreach had serious consequences. As a Kakute villager noted:

But for us when Ebola was suspected we kept expecting the health teams, but they did not turn up until a week later. So we thought that they did not care about us because for a whole week after the outbreak there was no health personnel to inform the people on how to go about it…

She argued that this perceived lack of concern and information from health workers escalated the idea that it was *amayembe*: “So I think if they had come earlier the health workers would have had less difficulty convincing the community about Ebola.”

The health system may have been slow to respond for several reasons. There is the practical challenge of getting samples, confirming lab results, and assembling and coordinating the response team and necessary logistics. One of our health worker respondents noted that there appeared to be conflict among the leadership over who was in charge. She also said it was difficult to persuade her colleagues to take the disease seriously:

I really felt that they were not serious. At that time we thought that the disease was Marburg and were very worried and anxious to know the results. Our colleagues from other sections had begun to distance themselves from us. We needed the results to know what we were dealing with, but it took them a whole week to give us the results after they had taken the samples. They took them on Thursday and told us on Wednesday. It did not please us that they were that lazy.

Respondents indicated that these delays were probably the result of insufficient funds to transport materials. However, the IFRC evaluation report noted that nobody wanted to drive the volunteers or lend them their transport, because outreach workers were seen as potential carriers, and people feared being associated with witchcraft [18]. When social mobilization did eventually start, the 20 volunteers sent door-to-door by the IFRC lacked sufficient material support, and experienced considerable anxiety regarding their own family contexts. In addition, they were not well acquainted with the wishes of the community. For example, they advised communities not to shake hands but instead to knock fist (*kubongo*), a practice locally associated with drug users and marginalized people. Moreover, the surveillance team appeared ill-trained in methods to assess the logic and needs of the local community. For example, Kabugo’s family neighbour, the same woman who had been the local yardstick for whether the disease was Ebola or *amayembe* (see above), was denied testing. As she herself said:

A team of health workers came to our village and took blood samples from selected people who were on their list. I told them to take my blood too, but they insisted that they were taking samples only from those who were on their list. My neighbour here was also tested.

While the interview does not tell us who made up this list, or what the criteria for selection were, ignoring the communal yardstick for Ebola illustrates a lack of sensitivity to the value of local knowledge and relationships. The IFRC evaluation report is strikingly focused on the patients and the medical system. When suspected cases were found, their isolated and even starving conditions were exclusively attributed to witchcraft. The report implied that it was witchcraft which caused “normal” social support elements (e.g. bringing food and water) at bay. When volunteers were asked about their motivation to volunteer “in such a setting”, the report notes that they felt it was “good to have the feeling that you were saving a life and keeping people safe by sensitizing the community who otherwise might have acted as far as killing the patients” [19] (P19). Focused mostly on the health of Ebola patients, volunteers, and health workers, the issue of community stigmatization or marginalization was addressed only at the level of economic marginalization. This discourse of framing witchcraft exclusively as a medical risk is also present in the accompanying IFRC strategy report, which explicitly noted that the disease required – through “culturally acceptable” interventions – “total behavior change from the community members (they still believe they are being attacked by witchcraft not Ebola) if the outbreak is to be controlled” [18] (p4). While biomedically accurate, this approach clearly failed to connect to local community health ideologies. The discourse in the reports is *against* witchcraft – and by extension, the community. This view that the community either needed change or was a barrier, instead of a necessary ally, appeared to be promoted in the media as well. For example, in an article on the Uganda Radio Network, the Luwero District Health Educator was quoted as having said that witchcraft allegations were “dangerous because they water down efforts to contain the deadly Ebola virus” [23].

### Mistrust of the health system

Kaguugo residents told us that the sensitization meetings failed to change their minds, and their interview narratives suggest that any contradiction in the meetings was seen as further evidence of a corrupt health system. For example, it had been announced on the radio that the government had released Ush600 million (approx. USD 175,900) to assist areas affected by Ebola, but local residents noted that they did not see any of this money. In Kakute, a respondent noted:

For us, we told them that we do not have Ebola, but they kept coming and we concluded that the health workers just wanted to eat the money that they had been given [laughs]. Yes. The truth needs to be told sometimes. The health workers came just for ceremonial purposes.

In a focus group held as part of sensitization meetings at Luwero town council during the outbreak, one of the respondents noted cynically that officials taught them how to prevent the spread of Ebola, but did not provide any water to wash their hands. Kaguugo community members asked why there been so little help from relief organizations despite calls for help by school heads. It was also noted more than once that schools were given just half a bottle of disinfectant to clean the entire school, and that no health camp had been set up near the community. And, as one focus group respondent pointed out, “These health workers would tell us not touch our patients who are ill, and at the same time expect us to deliver them to the health centers!?”

Many statements exemplify a sentiment of marginalization. People simply believed that while equipment and drugs were available, they were not helped because they were poor and unimportant. These words appear to have some truth in them. The surviving brother noted that some articles allocated to him and his brother-in-law at Mulago hospital – including new clothes and food items – never reached them. He discovered about these allocations through the health workers, who confirmed, on being queried, that they were supposed to have received these items.

Many locals remembered using an effective health care system in the past, but explained that they had become hostile to the system as the result of disillusionment with competition and bribery in government hospitals. To community members, this bribery was also entrenched in the much heralded community health worker system (VHTs) that consisted mostly of better off community members, typically including village chairs. Each village could pick one member to be sent for DTF training in Kampala, but community members argued that PLAN International would only select from the village leadership because they would hand out fees and thus control the agenda. Many conversations in offices and verandahs led to the conclusion that Ebola was an overblown conspiracy, developed to justify the allowances of medical personnel. Medical cases that seemed to counter the *amayembe* interpretation, like the survival of Kabugo’s brother and brother-in-law, were explained as the result of the corrupt health system itself. For rexample, according to the brother, some people thought that he and his mother-in-law had become rich as a result of the ordeal, as the radio had announced that the government of Uganda had given each Ebola patient three million Uganda shillings (approx. $860), which they were accused of pocketing.

While the community mistrusted the health system’s agenda, it also simultaneously experienced strong stigmatization from the outside world during this period, suffering serious economic consequences. As everyone was seen as contagious, the area had become a “danger zone”. A Kakute respondent said:

The discrimination was such that wherever we went, people would say that the people of Kakute village have Ebola. Even the people of Ndejje [a nearby community] did not want to pass through our road saying that there was Ebola at Kakute. You see? And even at the hospitals, the Kakute people were looked at with suspicion. They associated us with Ebola. The pregnant women who went for antenatal clinics suffered most, as the nurses told them to wait or sit aside as they treated others first.

As the idea of the contagion spread, so did the geographic isolation. In a sense, community members were right about *amayembe* contagion; now the entire Luwero area had become a danger zone of cultural stigma. Even health workers working within this community, helping the victims, were shunned by the public.

### The crisis of care

Local aetiologies, a discourse of community as barrier, and mistrust of the health system were major factors determining resistance, but it is equally, if not more, important to recognize that underlying this resistance was a local need for care. This need reached crisis proportions. A Kaguugo respondent explained:

My heart is not settled because I am used to other diseases that can allow your people to take care of you… But for this disease, you cannot take care of your people even when they are your father, your mother, your child or even your husband. You just run away from them! So the main question is ‘what should we do?’

Another Kakute resident noted that the inability to help a parent or someone close when in need “may haunt you forever”:

First of all she is my mother, and you are saying that touching her will also make me sick. So I think they have scared us too much, to the extent that one cannot even show love to their loved ones. In fact your mother can die cursing you if you don’t care for her when she is sick. She can say ‘my child refused to care for me when I was sick’ yet you haven’t really refused.

If one’s child catches Ebola, should a parent not touch to see what is happening? Would a parent run away from a child to save their own life? Should a neighbor not help the same people that helped her the last time she fell ill? The dilemma facing community members was that complying with quarantine requirements also introduced the risk of no longer being able to take care of loved ones:

I think they should calm down and tone down the scare. We know that the disease kills. I think they should also distribute the items which they tell people to buy like gloves, gumboots and soap. With those items people can take care of their relatives who develop the symptoms and even visit them at the Ebola clinic.

The Ebola control team collected all those who had been close to Ebola victims to monitor them in an Ebola isolation center. The IFRC evaluation describes how suspected cases –children or adults – were whisked away to health facilities, where they arrived potentially frightened and disoriented, “taken out of the car and sprayed with disinfectant and their possessions burnt in front of their eyes” (p15) [19]. Luwero respondents noted that within the emergency facilities, water and electricity were not available at first, while another prominent issue was the lack of food to sustain patients. According to a registered nurse working at the health center, local community members were generally not allowed to attend to their kin inside this center other than a brief glance.

This inability to provide care was present not only in terms of attendance, but also at communal level during burial rites. During a focus group with community members, the experience of burials appears to have been the most traumatic and visible violation of their community identity, leading to several incidents, involving the local Nubian community in particular: “They just dump them like dogs and return running fast from the burial ground like it is done for those who have committed suicide; that’s the way they do it.” There were also problems with cases which may not have been Ebola-related, but were also hurriedly dispatched. As the surviving brother noted, these disrespectful practices were relatively unnecessary:

For us Muslims, we have a special cloth to wrap the bodies in and the Christians use bark cloth. I think that if they didn’t want us to touch the bodies they would have asked us to buy these items so that our loved ones are wrapped and buried with the respect and in the clothes that we traditionally use for burial. They also should not just dump them. I saw them personally swing the body of a person and dump it on the vehicle.

According to our main medical informant, social mobilization “made them appreciate the reasons as to why we wanted the burial conducted that way.” Claiming that the subsequent burials were then handled in the right fashion, the respondent also noted in the same interview that the leaders within the burial group were not Muslims, instead referring to the affected, discordant group as “fanatics.” Overall the community felt that they had been made irrelevant in the struggle against Ebola. It appeared that one of the few places that had remained unaffected by the Ebola crisis was the local church, where residents were still allowed to convene. Ironically, the same spirituality that was heavily denied by authorities when it came in the form of *amayembe*, was here allowed to provide spiritual guidance and care. As a Kakute resident summarized it:

They told the people not to shake hands, not to converge, and schools were even closed because they did not want us to be in groups. But we used to go to the churches and mosques. These were allowed. We went there because God is the one who can deal with any situation including Ebola. So there we had no option but to seek God’s intervention.

## Conclusions

To solve the first mile problem, public health managers are quick to involve community health workers and social mobilization teams, ideally equipping them with smartphones to map the outbreak in real time. This study shows that in a major Ebola outbreak control effort in central Uganda in 2012, these interventions proved far from sufficient to assure collaboration from the community. Instead, the “people-centered approach” reaffirmed a historical mistrust between “scientific” biomedical and local explanations of illness and misfortune. This theme goes back as far as Evans-Pritchard’s *Witchcraft, Oracles and Magic among the Azande* (1937) [24] and is the subject of a truly immense body of anthropological literature. It illustrates the lack of credibility (from an emic perspective) of biomedical explanations that ignore local understandings, combined with the relatively logical and empirical basis of local explanations.

While biomedicine offered no clear evidence of Ebola as an infectious pathology, and no explanation as to why this outbreak occurred suddenly in this particular location and affected these particular individuals, the emic explanatory model involving *amayembe* was easily able to account for this by showing that the deaths had occurred in only one family, and that they involved people whom Ruth – who was accused of using the *amayembe* to bewitch them – did not like. Moreover, many people had had contact with the first patient, Kabugo, during his initial illness and during his funeral, but had not become infected. From an emic perspective, the evidence was clear.

While biomedical information appeared to be emitted into the community in relatively arbitrary and discrete bursts, these local interpretations were continuous and based on a sort of collective and cumulative empiricism, with local experiences, gossip and discussion feeding into and supporting an increasingly clear picture of what was “really” going on. People were proactive in collecting evidence to support their suspicions, for example by monitoring the woman who lived close to the Ebola victims and interacted with them, to see whether she also became infected.

The credibility of biomedical explanations was further damaged by the lack of fit between the general information that was circulated and the local situation on the ground. For example, the advice to avoid wild monkey meat might have been based on campaigns in areas where previous outbreaks occurred, and where this practice was common, but in an area where people did not eat monkey (and where there aren’t even monkeys to speak of) it did not inspire confidence in the veracity of the information. Similarly, the advice to use gloves and goggles when dealing with Ebola victims was both impractical (people have no access to these resources) and socially and culturally unacceptable as a way of caring for both sick and deceased relatives.

Although lack of funding, training and capacity appeared to have played a role, what characterizes this case study is the shared experience of community members that the DTF and its partners lacked openness and willingness to take the time needed to listen to and empathize with community needs. After all, what these community members were seeking first and foremost were strategies to take care of their fellow human beings and to diagnose and explain within their own cultural framework. The resistance to openness may be related to a pre-existing assumption that openness to other interpretations is the same as “accepting” them, and would automatically lead to a lack of control.

We acknowledge that the *amayembe* paradigm is difficult to reconcile with the biomedical view. Yet, ideas about *amayembe* do not necessarily exclude prompt treatment-seeking, a finding corroborated elsewhere [25]. Local practices and aetiology did motivate isolation and social control of those who were most likely to be carriers of the disease, because they were also, to an extent, the ones suspected of using the *amayembe*. Moreover, the interpretation of *amayembe* is not as uniform and static as might appear at first sight. Rather, we document a community discussion in which there is room for interpretation, doubt, and different views which may be held simultaneously. The existence of a local medical system characterized by resilience and syncretism is an observation supported by well-established anthropological facts [26]. It seems, therefore, counterproductive to deny this process.

Ironically, as the village and Luwero district became increasingly stigmatized and isolated by the outside world, the spread of *amayembe* from one family out into the community and district became its own case of social contagion that mimicked Ebola, but rested on different actors and different explanatory frameworks. While the outbreak in Luwero was ultimately controlled by an authoritative stance, one might wonder if this was helpful only in the short-term, as reemergence of the disease may only reinforce a divide with long historical roots. To be truly effective in bridging the first mile outbreak control, we believe respect and engagement are needed first and foremost, and despite remarkable technological innovations including real-time community information systems, these interventions remain contingent upon human interaction and openness to cultural difference. To develop a more “people-centered” early detection system, collaboration with the community must be at its core, and ideally the community should be seen as a resource instead of a barrier. To achieve this, the big missing element is knowing how to respectfully “agree to disagree” while simultaneously finding commonalities in humanity to facilitate collaboration during the first mile of outbreak control.

Given the mass of anthropological literature on traditional African aetiologies, and anthropological knowledge on a previous Ebola outbreak in Northern Uganda [16], it is surprising that so little serious effort was made to take local sensibilities and culture into account. It beggars belief, given this knowledge base and the extent of commonality in local aetiologies across sub-Saharan Africa, that this was largely ignored in the early response to a major epidemic in West Africa. Consequently, the “first mile” problem is not only a question of using local resources for early detection, but also of making use, in public health outreach, of the contextual cultural knowledge that has already been collected and is readily available.

These observations underscore the crucial importance of initiatives such as the Ebola Response Anthropology Platform^1^ aimed at providing access to regional and international rapid-response anthropological experts who could advise health authorities and governments as soon as an outbreak is suspected. This could also apply to other infectious diseases, because the underlying principles are the same. Another large body of literature which is relevant for explaining the distrust of the formal health system, and which also tends to be ignored when prevention campaigns do not work, is that which explores rumours and local distrust of “outsiders” perceived to be powerful and a potential threat to local interests. These may be “foreign” medical researchers thought to be stealing local people’s blood under the cover of clinical trials, or national health authorities secretly sterilizing women in the guise of immunisation campaigns, or disinformation campaigns deliberately intended to mislead people about the “real” cause of a disease [27, 28].

## Abbreviations

DTF: District task force
IFRC: red cross and red crescent
VHFs: viral hemorrhagic fevers
VHT: village health team

## Footnotes

1 http://www.ebola-anthropology.net/
